# Association between F‐box‐only protein 43 overexpression and hepatocellular carcinoma pathogenesis and prognosis

**DOI:** 10.1002/cam4.5660

**Published:** 2023-01-29

**Authors:** Shaohan Wu, Lei Qin, Juqin Yang, Jing Wang, Yiyu Shen

**Affiliations:** ^1^ Department of General Surgery The Second Affiliated Hospital of Jiaxing University Jiaxing China; ^2^ Department of General Surgery, Hepatobiliary Surgery The First Affiliated Hospital of Soochow University Suzhou China

**Keywords:** FBXO43, hepatocellular carcinoma, overexpression, prognosis

## Abstract

**Background:**

Despite great advances in the prevention, diagnosis, treatment, and management regarding hepatocellular carcinoma (HCC), the overall prognosis of HCC remains unfavorable. The expression profile, prognostic role, and biological functions of F‐box‐only protein 43 (FBXO43) in HCC remain unclear. Here, we determine the expression profile and prognostic value of FBXO43 in patients with HCC.

**Materials and Methods:**

A total of 467 HCC patients and their clinicopathological data were collected from the Second Affiliated Hospital of Jiaxing University, the Cancer Genome Atlas (TCGA), and Genotype‐Tissue Expression (GTEx) databases. The expression profile, prognostic value, biological functions, and underlying mechanism of its involvement of *FBXO43* were explored based on TCGA, Gene Expression Omnibus (GEO), LinkedOmics, and Cancer Dependency Map (DepMap). The expression of FBXO43 in 93 paired liver tissues was investigated via immunohistochemical staining, tissue microarray analysis, and Western blot. The prognostic value was assessed using survival analysis.

**Results:**

*FBXO43* RNA was upregulated in HCC liver tissues and was associated with an unfavorable prognosis (*p* < 0.05). Furthermore, FBXO43 protein was overexpressed in HCC liver tissues compared with that in paired normal liver tissues. Overexpression of FBXO43 protein was significantly associated with advanced TNM stage, large tumor size, lymphatic invasion, distant metastasis, earlier cancer recurrence, and decreased overall survival after radical surgery (*p* < 0.05). Cox regression analysis showed that FBXO43 had significant prognostic value in HCC. Importantly, *FBXO43* and its co‐expressed genes were mainly involved in cell cycle regulation, DNA replication, metabolic regulation, and so on. *FBXO43* knockdown could significantly affect the HCC cell lines growth and proliferation.

**Conclusions:**

We first revealed that *FBXO43* was overexpressed in liver HCC tissues at the RNA and protein levels and served as an independent prognostic factor for HCC patients. Therefore, *FBXO43* is worth investigating as a potential HCC treatment target.

## Graphical abstract

This article revealed that FBXO43 is overexpressed in HCC liver tissues at the RNA and protein levels and served as an independent prognostic factor for patients with HCC.

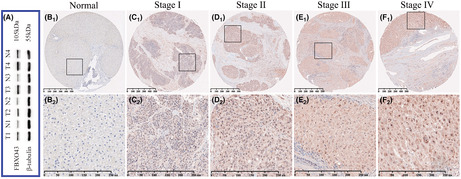

## 1 INTRODUCTION

Liver cancer, as a kind of common malignant tumor, is characterized by a high morbidity and mortality around the world.^1^, ^2^ China has the fourth‐highest morbidity and second‐highest mortality of liver cancer worldwide, according to the latest national cancer statistics. Hepatocellular carcinoma (HCC) is the main histological subtype, accounting for approximately 90% of primary liver cancers.^3^ HCC imposes a huge economic burden on both countries and patients. Although clinical diagnosis and treatment methods for HCC are constantly improving,^4^, ^5^, ^6^ the overall treatment effect is relatively unfavorable, as HCC is highly malignant and has high rates of metastasis and recurrence.^7^ Therefore, HCC remains an urgent global issue. Exploring the underlying pathogenesis, potential therapeutic targets, and key prognostic factors is imperative to facilitate reasonable prevention, early diagnosis, precise treatment, and effective management of HCC.^8^, ^9^

Various genes and biomarkers that are downregulated or upregulated in patients with HCC are considered prognostic factors or therapeutic targets.^10^, ^11^ The F‐box gene family exhibits excellent potential for therapeutic applications in human cancers, including liver cancer, and plays important roles in cellular biological processes.^12^, ^13^, ^14^, ^15^ Most members of the F‐box protein (FBP) family are involved in DNA damage repair, cell cycle regulation, metabolic regulation, and other biochemical processes.^13^, ^14^, ^15^, ^16^ These biological processes are closely associated with tumors. FBP gene family mainly includes FBXL, FBXO, and FBXW subfamilies. More than 37 FBXO members have been identified.^15^ Among them, the translation products of F‐box‐only protein 43 (FBXO43) belong to the FBP family and share the structural characteristics of the F‐box protein,^17^ which may enable FBXO43 to play a significant role in the development and occurrence of tumors. Early mitotic inhibitor 2 (EMI2), a translation product of *FBXO43*, is an unfavorable prognostic biomarker in breast cancer patients.^18^ Moreover, *FBXO43* RNA is significantly overexpressed at the transcription level in HCC and gastric cancer (GC).^19^ *FBXO43* RNA was found to be a poor prognostic factor of HCC based on a gene co‐expression network analysis in 2019.^8^ Up to now, few studies have detailed the role of *FBXO43* in malignant tumors other than breast cancer.^12^, ^18^ In particular, at the transcriptional and translational levels, the association between *FBXO43* and the pathogenesis and prognosis of HCC remains poorly understood, requiring further exploration.

In this study, we aimed to determine the expression profile and prognostic role of *FBXO43* through bioinformatics analysis and then verified the findings in clinical samples. Moreover, we preliminarily investigated the biological functions and the underlying mechanism of its involvement of *FBXO43*. The expression of FBXO43 RNA and protein was significantly increased in HCC liver tissues and predicted an unfavorable prognosis in HCC patients. These results highlight the clinical significance of *FBXO43* in the prognosis of patients with HCC.

## 2 MATERIALS AND METHODS

### 2.1 Data and liver tissues collection

A total of 374 HCC patients were identified in The Cancer Genome Atlas (TCGA) database (https://portal.gdc.cancer.gov/). Then, we downloaded RNA sequencing data and corresponding clinical data for HCC tissues (*n* = 374) and adjacent normal liver tissues (*n* = 50). The follow‐up time for subjects in different researches collected from TCGA database was not same. The follow‐up time of many patients was more than 10 years. We also downloaded gene expression data of normal liver tissues (*n* = 175) in Genotype‐Tissue Expression (GTEx) database (http://commonfund.nih.gov/GTEx/). Therefore, 225 normal liver tissues were included in the study. Microarray datasets of GSE101685, GSE101728, GSE112790, GSE25097, GSE29721, GSE33006, GSE50579, GSE54238, GSE6222, GSE62232, GSE64041, and GSE89377 were downloaded from the Gene Expression Omnibus (GEO) website (https://www.ncbi.nlm.nih.gov/geo/). The inclusion criteria of GEO datasets mainly included the gene expression profiles of *FBXO43* in liver hepatocellular carcinoma (LIHC; namely HCC) and normal liver tissues, data type (RNA sequencing), analysis level (gene), and species (human origin). The exclusion criteria mainly included the small sample size (*n* < 6) or lack of the expression data of *FBXO43* or invalid data that could not be analyzed under existing conditions. TCGA, GTEx, and GEO databases were available freely. The local ethical approval was not required.

HCC tissues (*n* = 93) and adjacent normal tissues (*n* = 93) were retrospectively collected in the Second Affiliated Hospital of Jiaxing University between 1 January 2014 and 1 November 2017. The diagnoses were verified by two skilled pathologists who were blinded to the patient data. Demographic and clinicopathological data were retrospectively collected, including preoperative alpha‐fetoprotein (AFP) level, sex, tumor number, age, tumor distribution, tumor tumor–nodule–metastasis (TNM) stage, tumor size, tumor differentiation, and recurrence. Patients were followed up until 31 January 2022. Among the 93 patients from our hospital, 24 patients were lost to follow‐up, resulting in a rate of lost to follow‐up of 25.8%. Overall survival (OS) and disease‐free survival (DFS) were the two study endpoints, defined respectively as the time interval between the first radical resection and death and as the time interval between the first radical resection and HCC recurrence or metastasis. All patients met all of the following criteria: presence of primary HCC confirmed by histological and clinical pathological results, age between 18 and 80 years, Chinese Han ethnicity, and no radiotherapy, chemotherapy, immunotherapy, targeted therapy, or other treatment received before radical surgery. Patients who met any of the following criteria were excluded: incomplete pathological data or medical records; presence of very serious diseases of the heart, lungs, or other important organs; and pregnancy. All patients involved in this study presented written informed consent. This study was implemented based on the Declaration of Helsinki and its amendments and was approved by the Ethical Committee of the Second Affiliated Hospital of Jiaxing University (Ethical Committee number: JXEY‐2021JX147). Liver tissues were immediately cryopreserved in liquid nitrogen and then refrigerated at −80°C until protein extraction was performed.

### 2.2 Expression profile of *FBXO43* at mRNA level in TCGA, GTEx, and GEO databases

The edgeR package in R software was used to convert the counts data of the *FBXO43* into counts per million (CPM), using qCML method. We determined P‐values based on the negative binomial distribution combined with Fisher's exact test. Anova package of R software was utilized to identify the expression level of *FBXO43* RNA in HCC tissues among four pathological stages. The expression patterns of *FBXO43* mRNA in TCGA and GTEx databases were analyzed by Wilcoxon rank sum test in R software (version 3.6.3). *FBXO43* expression in the GEO datasets was analyzed using the limma package in R software.

### 2.3 The prognostic value of FBXO43 mRNA in TCGA database

The survival differences including Kaplan–Meier analysis and the log‐rank test, Cox regression analysis, hazard ratio (HR) of the Cox proportional hazard regression model, and p‐value were calculated and analyzed using the “survival” and “survminer” package in R software.

### 2.4 Protein extraction and Western blot (WB) analysis

Total protein was extracted from four pairs of HCC tissues and matched normal tissues using a whole‐protein extraction kit (Beyotime Biotechnology, Shanghai, China). Then, 30 μg protein samples were transferred to polyvinylidene difluoride membranes (Millipore, MA, USA). The membranes were fully washed, blocked, and incubated with anti‐FBXO43 primary antibodies (https://www.thermofisher.cn/cn/zh/antibody/product/FBXO43‐Antibody‐Polyclonal/PA5‐21622, Invitrogen) and β‐tubulin (Proteintech) overnight at 4°C, and then incubated with appropriate horseradish peroxidase‐conjugated secondary antibodies (Abcam). Finally, signals of FBXO43 and β‐tubulin protein were detected using enhanced chemiluminescence (Pierce Biotechnology).

### 2.5 Tissue microarray (TMA) analysis

All HCC specimens were separately fixed with 10% paraformaldehyde and embedded in paraffin blocks. Then, they were sectioned consecutively at a thickness of 4 mm. Ninety‐three pairs of HCC samples and matched normal tissues were constructed for TMA analysis, as we previously described.^9^

### 2.6 Immunohistochemistry (IHC) analysis

Tissues were incubated overnight at 4°C with anti‐FBXO43 primary antibody (Invitrogen) followed by incubation with a secondary antibody (Golden Bridge Biotechnology Co.) for 30 min at room temperature. FBXO43 protein staining was scored independently by two experienced pathologists who were blinded to the patients' information, as previously described.^20^

### 2.7 Linkedomics database analysis

Based on LinkedOmics database (http://www.linkedomics.org/login.php), FBXO43 co‐expressed genes in HCC were investigated using Pearson's correlation coefficient analysis and displayed as volcano plot and heat maps. The Gene Set Enrichment Analysis (GSEA) for Gene Ontology (GO) terms and Kyoto Encyclopedia of Genes and Genomes (KEGG) pathways were analyzed by the “LinkInterpreter” module of LinkedOmics. GO terms included BP (biological processes), CC (cellular components), and MF (molecular functions). The false discovery rate (FDR) for the rank criterion was less than 0.05, and simulations were 1000.

### 2.8 Cancer dependency map (DepMap) database analysis

DepMap online tool was used to visualize the data from the Cancer Cell Line Encyclopedia (CCLE) database. DepMap (https://depmap.org/portal/) combines Clustered Regularly Interspaced Short Palindromic Repeats (CRISPR) and RNA interference (RNAi) data and is applied to determine gene dependencies of human genes in numerous cancer cell lines. The gene effect score of individual gene is obtained from screening experiments. The scores appraise the effect size of knocking down or knocking out human genes. A negative score indicates that the cell lines grow slower after knocking down or knocking out of a gene, while a positive score indicates that the cell lines grow faster.^21^ Seven common HCC cell lines were analyzed, including SNU387 and HEPG2.

### 2.9 Statistical analysis

There are two sets of survival calculation in this study, one was from clinical series (the Second Affiliated Hospital of Jiaxing University), the other one was from the public database, with different “start dates” of enrolling the observation cohort. The follow‐up time of many patients in the TCGA database were more than 10 years. The raw data from TCGA, GTEx, and GEO were analyzed by R software and corresponding packages. The data collected from clinical research in our hospital were analyzed using the SPSS 23.0 statistical software package (SPSS Inc.). The Chi‐square (*χ* ^2^) test or Student's *t*‐test (two‐sided) was applied to determine the association between FBXO43 expression and the clinicopathological and demographic parameters of HCC patients. DFS and cumulative OS were plotted using Kaplan–Meier analysis and the log‐rank test based on different datasets respectively. The Cox proportional hazard regression models were utilized to conduct univariate and multivariate regression analyses. Statistical significance was set at *p* < 0.05.

## 3 RESULTS

### 3.1 Expression profile of *FBXO43* RNA in 35 malignant tumor types in TCGA database

**FIGURE 1:**
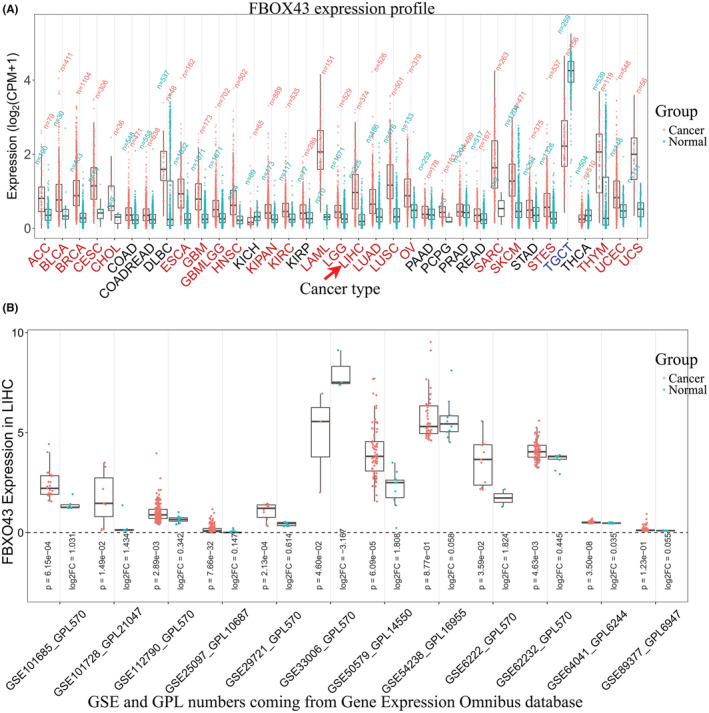
Upregulated expression of *FBXO43* RNA in most malignant tumors including HCC based on bioinformatics analysis. (A) In TCGA database, in 23 types of malignant tumors (red font) including LIHC (red arrow), *FBXO43* RNA was overexpressed in tumor tissues compared with that in matched normal tissues (*p* < 0.05). (B) In 10 out of 12 datasets in the GEO database, *FBXO43* RNA was upregulated in HCC liver tissues compared with that in normal liver tissues (*p* < 0.05). FBXO43, F‐box‐only protein 43; GEO, Gene Expression Omnibus; GSE, GEO series; GPL, GEO platform; HCC, hepatocellular carcinoma; LIHC, liver hepatocellular carcinoma; TGGA, The Cancer Genome Atlas.

Thirty‐five malignancies involving *FBXO43* RNA expression were included. Surprisingly, *FBXO43* RNA was significantly overexpressed in tumor tissues compared to that in matched normal tissues in 23 malignancies, including liver HCC (Figure 1A, *p* < 0.05). *FBXO43* RNA was downregulated only in testicular germ cell tumors (Figure 1A, *p* < 0.05). There was no striking difference in other 11 types of malignant tumors (Figure 1A, *p* > 0.05). These results revealed that *FBXO43* RNA expression was upregulated in most malignancies.

### 3.2 Overexpression of *FBXO43* RNA in HCC tissues in the GEO database

We included 12 GEO datasets named after the GEO Series (GSE) number and GEO platform (GPL) number. Each dataset can be accessed on the GEO website. We then analyzed the expression profile of *FBXO43* RNA. In 10 datasets, *FBXO43* RNA was highly expressed in HCC tissues (Figure 1B, *p* < 0.05). The GSE54238 and GSE89377 datasets showed no significant differences (Figure 1B, *p* > 0.05).

### 3.3 Overexpression of *FBXO43* RNA and poor clinical outcomes in HCC patients in TCGA database

**FIGURE 2:**
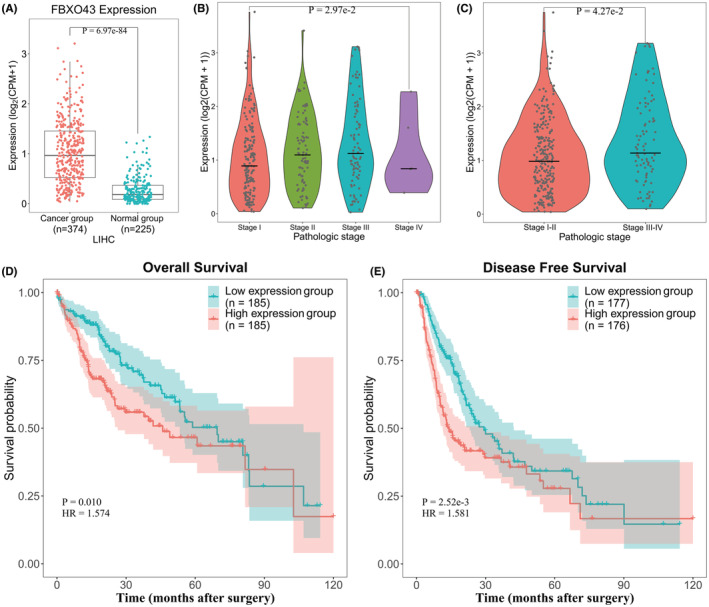
Upregulated expression of *FBXO43* RNA in HCC liver tissues and its influence on survival time based on TCGA data. (A) *FBXO43* RNA expression in the cancer group, which included 374 HCC liver tissues, was upregulated compared with that in the normal group, which included 225 normal liver tissues (*p* = 6.97 e‐84). (B) There was a significant difference in expression according to pathological stage (*p* = 2.97 e‐2). From stage I to stage III, the expression of *FBXO43* RNA gradually increased. (C) The expression of *FBXO43* RNA in the stage III/IV group was much higher than that in the stage I/II group (*p* < 0.0427). (D) High expression of *FBXO43* RNA was associated with an unfavorable OS (*p* = 0.001). (E) High expression of *FBXO43* RNA was associated with poor DFS (*p* = 2.52 e‐3). FBXO43, F‐box‐only protein 43; HCC, hepatocellular carcinoma; RNA, ribonucleic acid; TGGA, The Cancer Genome Atlas.

The expression of *FBXO43* RNA in HCC tissues was significantly increased compared to that in normal liver tissues (Figure 2A, *p* = 6.97 e‐84). Furthermore, there were significant differences among tumors in the four pathological stage (Figure 2B, *p* = 2.97 e‐2). From stage I to stage III, the expression of *FBXO43* RNA in HCC tissues was gradually upregulated. Although the expression of *FBXO43* RNA in stage IV significantly decreased compared with that in stages II and III, there were only five samples in the stage IV group, which might have resulted in bias. Furthermore, there was a significant difference between stages I‐II and stages III‐IV (Figure 2C, *p* = 4.27 e‐2). These results suggest that the expression of *FBXO43* RNA in HCC tissues may gradually increase as HCC progresses.

Next, we evaluated the relationship between *FBXO43* RNA expression and DFS and OS in patients with HCC. In terms of clinical data of HCC patients in TCGA, only 353 of 374 patients obtained DFS data and only 370 patients obtained OS data. Patients with incomplete clinical data were excluded. Based on the median expression value of *FBXO43* RNA, 353 HCC patients with DFS data and 370 HCC patients with OS data were divided into high and low expression groups. All DFS and OS data were used for Kaplan–Meier survival analysis. A significant difference in the OS and DFS was observed between the high and low expression groups. The OS and DFS in the high expression group were much shorter than those in the low expression group (Figure 2D,E, *p* = 0.01 for OS. *p* = 2.52 e‐3 for DFS).

**FIGURE 3:**
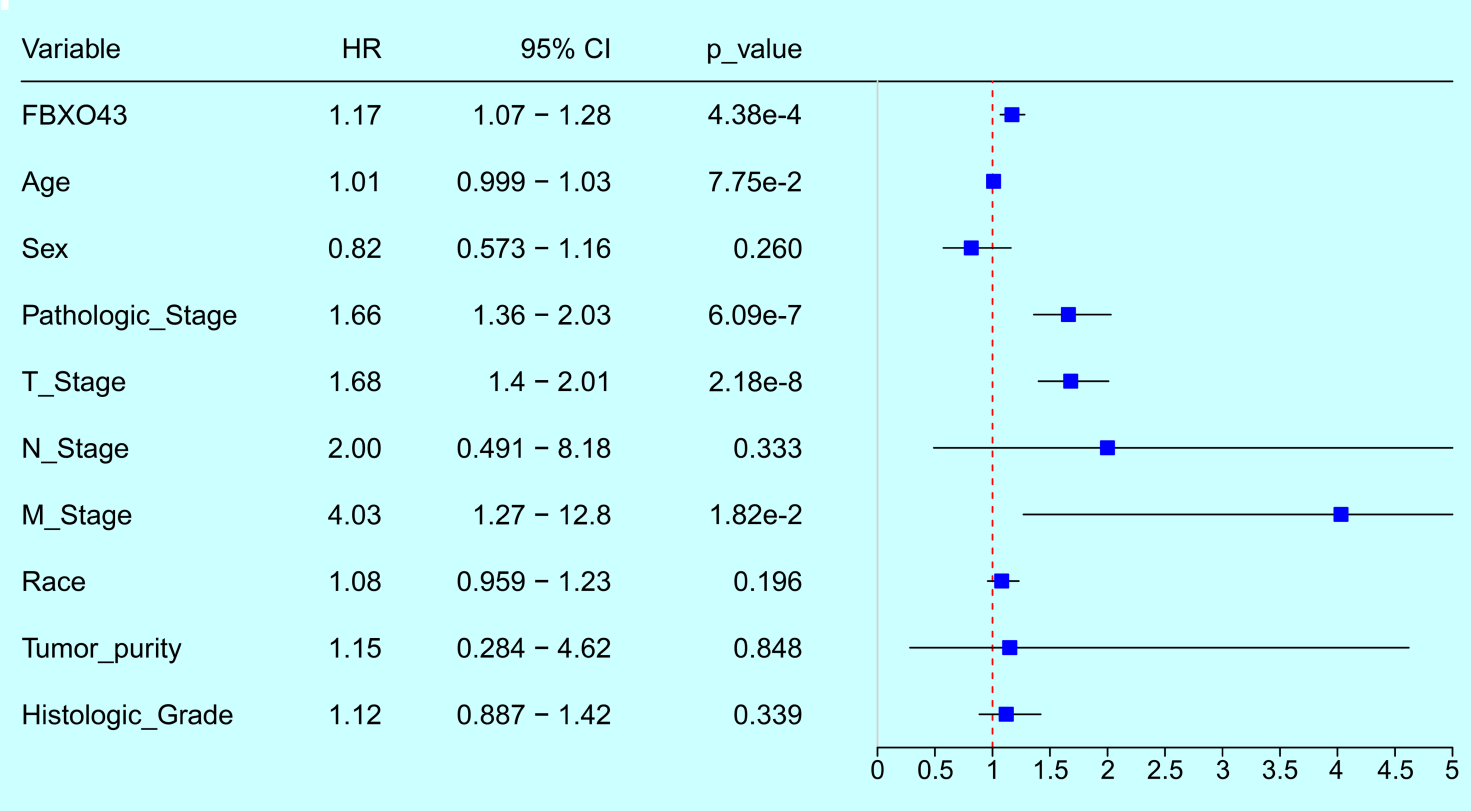
Overexpression of FBXO43 predicted poor survival in patients with HCC collected from TCGA database. Based on TCGA data, high expression of *FBXO43* RNA, advanced pathological stage, T stage, and distant metastasis were hazardous factors in HCC patients (*p* < 0.05). 95% CI, 95% confidence interval; FBXO43, F‐box‐only protein 43; HCC, hepatocellular carcinoma; HR, hazard ratio; RNA, ribonucleic acid; TNM, tumor nodule metastasis.

In univariate Cox regression analysis, the HR in the high expression group was significantly higher than that in the low expression group (Figure 3, HR = 1.17, 95% CI: 1.07–1.28, *p* = 4.38 e‐4). Additionally, the pathological stage, tumor (T) stage, and metastasis (M) stage were more advanced in the high expression group (Figure 3, HR > 1, 95% CI >1, *p* < 0.05, respectively).

### 3.4 Elevated expression of FBXO43 in HCC tissues at the translational level

**FIGURE 4:**
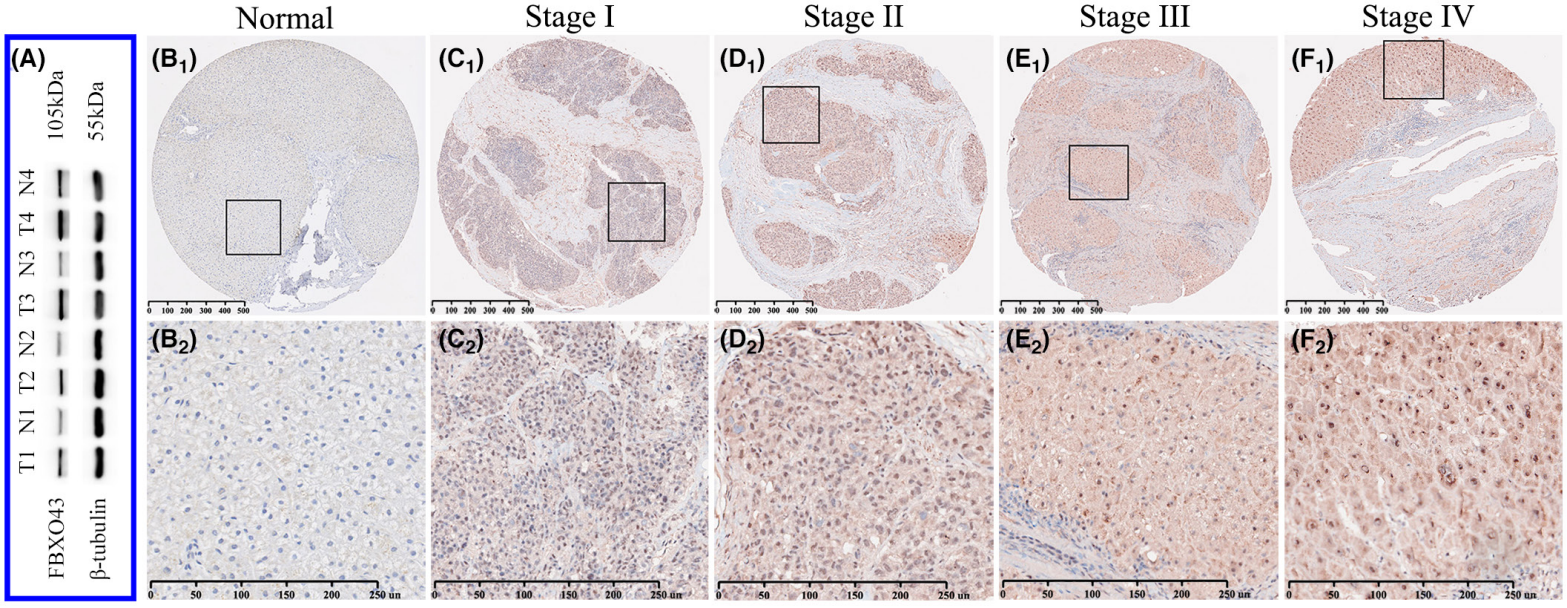
Expression of FBXO43 protein in HCC tissues was higher than that in paired normal liver tissues. (A) Western blot results revealed overexpression of FBXO43 protein in 4 liver tumor tissues. (B_1_ and B_2_) Negative FBXO43 expression in paired adjacent normal liver tissues. (C_1_ and C_2_) Weak expression of FBXO43 in HCC tissues. (D_1–2_ and E_1–2_) Positive expression of FBXO43 in HCC tissues. (B–E) Expression of FBXO43 increased as HCC progressed to more advanced AJCC stages. AJCC, American Joint Committee on Cancer; FBXO43, F‐box‐only protein 43; HCC, hepatocellular carcinoma.

IHC staining for FBXO43 protein was preliminarily performed with five pairs of HCC liver tissues and adjacent normal liver tissues. FBXO43 protein was located in both the nucleus and cytoplasm of the hepatocytes. Most of liver specimens exhibited positive expression (data not shown). WB analysis verified that FBXO43 protein was distinctly overexpressed in HCC tissues compared with that in paired normal liver tissues (Figure 4A). We then used TMA to determine the expression of FBXO43 in 93 human HCC specimens and adjacent normal tissues. Similar to the IHC staining results, the FBXO43 protein was mainly located in both the nucleus and cytoplasm of the hepatocytes. The FBXO43 protein was not highly expressed in the 93 pairs of liver tissues. In HCC tissues, staining for the FBXO43 protein was positive in 61 (65.6%) and negative in 32 (34.4%) samples, whereas in matched normal tissues, staining was positive in 39 (41.9%) and negative in 54 (58.1%) samples (Table 1, *p* = 0.001). Therefore, compared with that in paired normal liver tissues, FBXO43 was significantly upregulated in HCC liver tissues. Additionally, the expression level of FBXO43 increased significantly as HCC progressed to more advanced pathological stages (Figure 4B_1–2_–F_1–2_).

**TABLE 1:** Expression of FBXO43 protein in HCC tissues and paired normal tissues.

Tissue sample	Number	FBXO43 expression		*p* Value
		Negative/Weak (%)	Positive (%)	
HCC tissues	93	32 (34.4%)	61 (65.6%)	**0** **.001**
normal tissues	93	54 (58.1%)	39 (41.9%)	

*Note*: Bold indicates the values *p* < 0.05 are statistically significant.

Abbreviations: FBXO43, F‐box‐only protein 43; HCC, hepatocellular carcinoma.

### 3.5 Association between FBXO43 protein expression in HCC tissues and clinicopathological parameters

According to the IHC scores, HCC liver tissues exhibiting positive expression of FBXO43 protein were included in the high FBXO43 expression group, and tissues exhibiting negative or weak expression were included in the low FBXO43 expression group. The association between the expression levels of FBXO43 protein and clinicopathological characteristics was assessed (Table 2). FBXO43 overexpression was strongly associated with larger tumor size, advanced tumor–nodule–metastasis (TNM) stage, lymphatic invasion, advanced T stage, and advanced N stage (Table 2, *p* < 0.05). Meanwhile, no correlations were observed between FBXO43 expression and sex, age, preoperative alpha‐fetoprotein (AFP) level, tumor number, tumor distribution, pathological M stage, and degree of tumor differentiation (Table 2, *p* > 0.05). At the translational level, these findings indicate that FBXO43 overexpression is related to the clinical progression of HCC. TNM stages were classified according to American Joint Committee on Cancer (AJCC).

**TABLE 2:** Relationship between the expression of FBXO43 and the clinicopathological characteristics in patients with HCC (*n* = 93).

Variables	Total No. of patients	High FBXO43 expression (*n*)	Low FBXO43 expression (*n*)	χ2 Value	*p* Values
Sex					
Female	34	16	18	0.681	0.409
Male	59	33	26		
Age (Year)					
<60	47	27	20	0.863	0.353
≧60	46	22	24		
Preoperative AFP level (ng/mL)					
≦20	37	17	20	1.121	0.290
>20	56	32	24		
Tumor number					
≦3	49	36	13	0.924	0.336
>3	44	36	8		
Tumor distribution					
Single	70	33	37	3.492	0.062
Multiple	23	16	7		
Tumor size (cm)					
≦5	71	32	39	6.987	**0.008**
>5	22	17	5		
Tumor TNM stage					
I–II	52	20	32	9.577	**0.002**
III–IV	41	29	12		
T stage					
T1–T2	71	33	38	4.642	**0.031**
T3–T4	22	16	6		
N stage					
N0	82	40	42	4.247	**0.039**
N1	11	9	2		
M stage					
M0	89	46	43	0.835	0.361
M1	4	3	1		
Tumor differentiation					
Well/Moderate	62	30	32	1.380	0.240
Poor	31	19	12		
Recurrence					
No	48	20	28	4.834	**0.028**
Yes	45	29	16		

*Note*: Bold indicates the values *p* < 0.05 are statistically significant.

Abbreviations: AFP, preoperative alpha‐fetoprotein; FBXO43, F‐box‐only protein 43; HCC, hepatocellular carcinoma; TNM, tumor nodule metastasis.

### 3.6 Prognostic significance of FBXO43 expression

**FIGURE 5:**
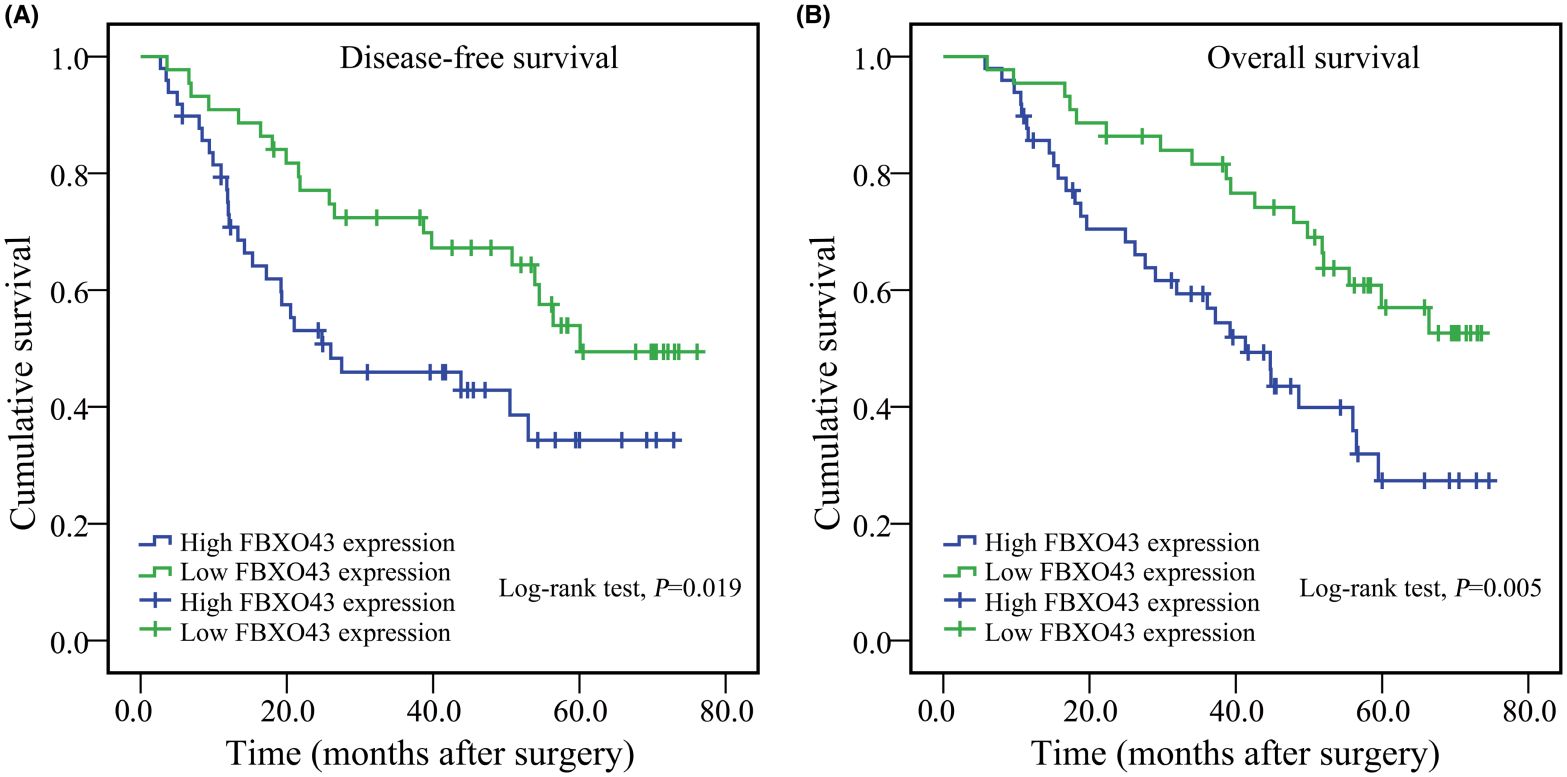
Overexpression of FBXO43 protein predicted poor DFS and OS in patients with HCC collected from our center. (A) High expression of FBXO43 protein predicted poor DFS in HCC patients (*p* = 0.019). (B) High expression of FBXO43 protein was associated with poor OS in patients with HCC (*p* = 0.005). DFS, disease‐free survival; FBXO43, F‐box‐only protein 43; HCC, hepatocellular carcinoma; OS, overall survival.

We further estimated the effect of FBXO43 expression at the protein level on DFS in patients with HCC. Compared with the low FBXO43 expression group, the high FBXO43 expression group exhibited a significant difference in DFS and OS. Higher expression of FBXO43 protein predicted much earlier recurrence (Figure 5A, *p* = 0.019) and decreased OS (Figure 5B, *p* = 0.005) after curative surgery.

Next, univariate regression analysis was conducted using the Cox proportional hazards model. Patients in the high FBXO43 expression group had a HR that was over twofold higher than that of patients in the low‐FBXO43 expression group (Table 3, HR = 2.533, 95% CI: 1.365–4.701, *p* = 0.003). This trend was observed in terms of OS, as well (Table 3, HR = 2.580, 95% CI: 1.411–4.717, *p* = 0.002). Furthermore, tumor size, tumor number, TNM stage, T stage, M stage, and degree of tumor differentiation were dramatically related to both DFS and OS (Table 3, *p* < 0.05, respectively).

**TABLE 3:** Univariate analyses of the clinicopathological characteristics associated with DFS and OS.

Variables	DFS		OS	
	Hazard ratio (95% CI)	*p* values	Hazard ratio (95% CI)	*p* values
Sex (Female vs. Male)	0. 950 (0.516–1.749)	0.869	0.945 (0.521–1.714)	0.852
Age, year (≧50vs. < 50)	1.222 (0.680–2.196)	0.502	1.375 (0.771–2.453)	0.280
Preoperative AFP level (ng/mL, >20 vs.≦20)	1.821 (0.964–3.440)	0.065	2.168 (1.142–4.117)	**0.018**
Tumor number (>3 vs.≦3)	2.187 (1.173–4.077)	**0.014**	1.949 (1.039–3.655)	**0.038**
Tumor distribution (Multiple vs. Single)	1.344 (0.715–2.528)	0.385	1.121 (0.591–2.124)	0.727
Tumor size (cm, >5 vs.≦5)	2.741 (1.473–4.903)	**0.004**	2.061 (1.112–3.821)	**0.022**
Tumor TNM stage (III–IV vs. I–II)	3.329 (1.791–6.190)	<**0.001**	2.297 (1.229–3.926)	**0.007**
T stage (T3–T4 vs. T1–T2)	1.713 (1.131–3.289)	**0.026**	1.496 (1.147–2.946)	**0.021**
N stage (N1 vs. N0)	1.531 (0.646–3.629)	0.334	1.375 (0.835–2.983)	0.256
M stage (M1 vs. M0)	17.256 (5.096–58.436)	<**0.001**	7.034 (2.403–20.589)	<**0.001**
Tumor differentiation (Poor vs. Well/Moderate)	2.278 (1.296–5.090)	**0.005**	2.133 (1.196–3.806)	**0.010**
FBXO43 expression (High vs. Low)	2.533 (1.365–4.701)	**0.003**	2.580 (1.411–4.717)	**0.002**

*Note*: Bold indicates the values *p* < 0.05 are statistically significant.

Abbreviations: AFP, preoperative alpha‐fetoprotein; DFS, disease‐free survival; FBXO43, F‐box‐only protein 43; OS, overall survival; TNM, tumor nodule metastasis; vs., versus; 95% CI, 95% confidence interval.

We hypothesized that FBXO43 was associated with distant metastasis and lymph node invasion. The small sample size in the N1 and M1 groups might have introduced an obvious bias, and result in significant differences in the univariate regression analysis regarding pathological M stage and N stage. Therefore, tumor number, TNM stage, histological differentiation, and the expression levels of FBXO43 were further evaluated by multivariate regression analysis. Ultimately, advanced tumor TNM stage, poor histological differentiation, and high expression of FBXO43 predicted a higher risk of decreased OS, and earlier carcinoma recurrence (Table 4, *p* < 0.05).

**TABLE 4:** Multivariate analyses of factors associated with DFS and OS.

Variables	DFS		OS	
	Hazard ratio (95% CI)	*p* values	Hazard ratio (95% CI)	*p* values
Tumor number (>3 vs. ≦3)	1.920 (1.109–3.660)	**0.042**	1.824 (0.909–3.705)	0.091
Tumor TNM stage (III–IV vs. I–II)	2.132 (1.325–3.574)	**0.016**	2.357 (1.204–3.976)	**0.009**
Tumor Differentiation (Poor vs. Well/Moderate)	2.101 (1.532–3.626)	**0.025**	1.682 (1.117–3.204)	**0.031**
FBXO43 expression (High vs. Low)	2.011 (1.242–3.882)	**0.037**	2.180 (1.157–4.105)	**0.016**

*Note*: Bold indicates the values *p* < 0.05 are statistically significant.

Abbreviations: DFS, disease‐free survival; FBXO43, F‐box‐only protein 43; OS, overall survival; TNM, tumor nodule metastasis; vs., versus; 95% CI, 95% confidence interval.

### 3.7 *FBXO43* is involved in different biological functions and signaling pathways

**FIGURE 6:**
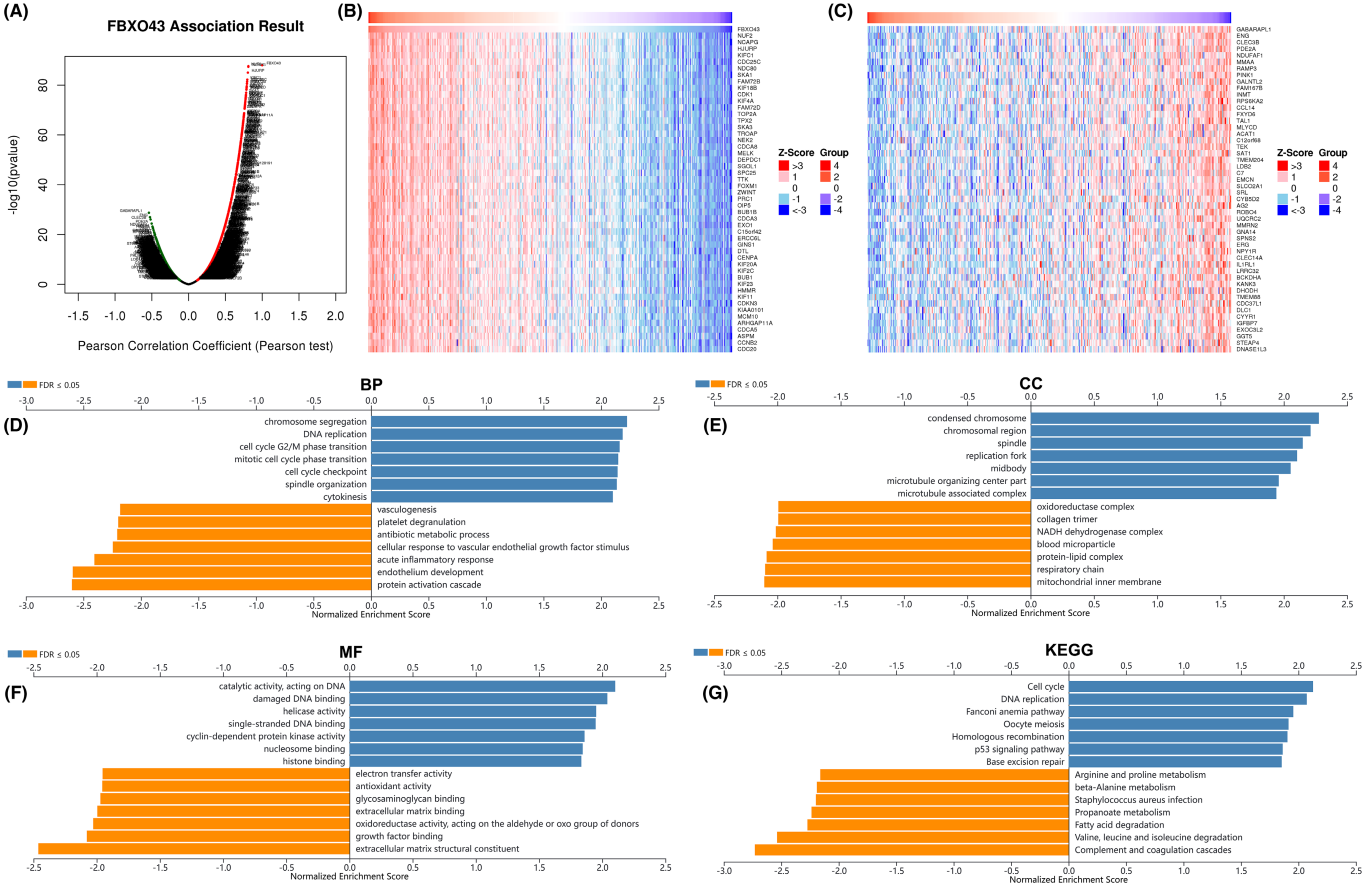
Prediction of biological functions and signaling pathways for *FBXO43* in HCC based on LinkedOmics database. (A) The volcano plot showed the correlation analysis between *FBXO43* and other genes in HCC. (B) The top 50 genes positively correlated with *FBXO43*. (C) The top 50 genes negatively correlated with *FBXO43*. (D–F) GO terms analysis for BP, CC, and MF, respectively. (G) KEGG pathway analysis. BP, biological processes; CC, cellular components; FBXO43, F‐box‐only protein 43; GO, Gene Ontology; HCC, hepatocellular carcinoma; KEGG, Kyoto Encyclopedia of Genes and Genomes; MF, molecular functions.

The biological functions of *FBXO43* were predicted by LinkedOmics. All the red dots were genes significantly positively associated with the *FBXO43* expression in HCC, and green dots were negatively associated genes (Figure 6A, *p* < 0.05). The top 50 genes positively and negatively associated with *FBXO43* were showed in the form of heat maps (Figure 6B,C, respectively). The top 3 genes positively co‐expressed with *FBXO43* were *NUF2*, *NCAPG*, and *HJURP*. Regarding BP, *FBXO43* and its co‐expressed genes mainly involved in chromosome segregation, DNA replication, cell cycle checkpoint, mitotic cell cycle phase transition, and cell cycle G2/M phase transition of cell division cycle (Figure 6D). Regarding CC, those genes mainly participated in condensed chromosome, chromosomal region, spindle, and respiratory chain (Figure 6E). Regarding MF, those genes mainly located in catalytic activity, acting on DNA, damaged DNA binding, single‐stranded DNA binding, helicase activity, and cyclin‐dependent protein kinase activity (Figure 6F). Regarding KEGG pathways, those genes mainly were implicated in cell cycle, DNA replication, Fanconi anemia pathway, homologous recombination, p53 signaling pathway, and so on (Figure 6G).

### 3.8 Dependency scores of *FBXO43* in HCC cell lines

**FIGURE 7:**
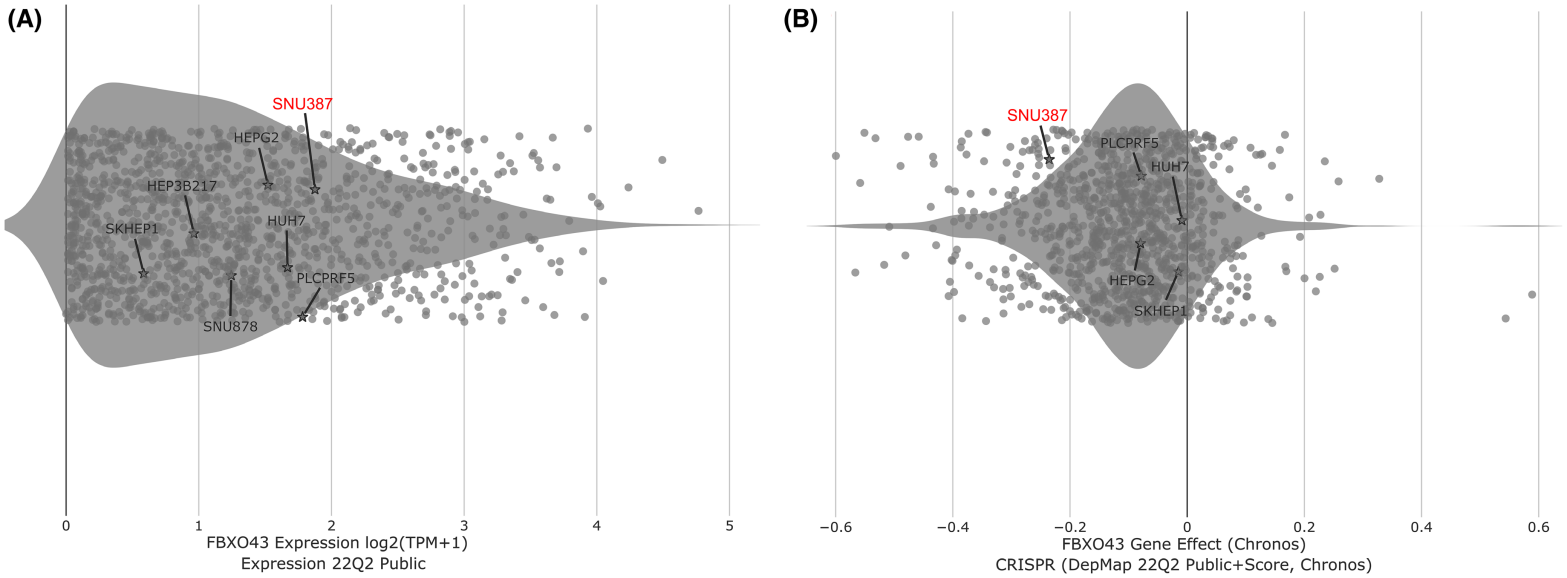
Summary of the expression characteristics and necessity of *FBXO43* in seven common HCC cell lines. (A) Based on “Expression” dataset, the summary of *FBXO43* expression. Each gray dot represents a cell line. (B) Based on “CRISPR” dataset, the perturbation effect of *FBXO43* knockdown on cell growth. A negative score indicates that the cell lines grow slower after knocking out of a gene, while a positive score indicates that the cell lines grow faster. CRISPR, clustered regularly interspaced short palindromic repeats; FBXO43, F‐box‐only protein 43.

We can obtain *FBXO43* expression patterns in multiple human HCC cell lines and potential effect of *FBXO43* knock out on cell growth and proliferation. The FBXO43 expression in log2 (TPM + 1) format is 1.876, and the perturbation effect (CRISPR) is −0.236 (Figure 7A,B, respectively). Considering the expression level and perturbation effect, *FBXO43* was an essential gene on the growth or survival of SNU387. *FBXO43* knockdown attenuates the growth of liver cancer cells.

## 4 DISCUSSION

In this study, we explored the expression profile of *FBXO43* and revealed the prognostic role in patients with HCC through bioinformatics analysis. Based on TCGA data, most malignant tumors, including HCC, *FBXO43* RNA expression was significantly higher expressed in tumor tissues than in normal liver tissues. Based on GEO data, *FBXO43* RNA increased in HCC tissues than in matched normal liver tissues. The results were consistent between the two databases. A recent study found a similar expression pattern in patients with HCC.^8^ Based on TCGA and GTEx data, *FBXO43* RNA expression in HCC group was much higher than that in the normal group. More importantly, the expression of *FBXO43* RNA gradually increased as HCC progressed to a more advanced pathological stage, except for stage IV which was represented by only five cases. The small sample size in stage IV group might cause significant bias. Moreover, high expression of *FBXO43* RNA was associated with a more unfavorable DFS and OS than low expression group. These results suggest that overexpression of *FBXO43* RNA in HCC liver tissues predict poor prognostic and clinical outcomes.

Regrettably, these specific results were obtained at the RNA level, and results at the protein level may differ. As we all know, gene expression is complex. Proteins execute the functions driving the occurrence and development of tumors, and the processes of gene transcription and translation are not completely consistent. In other words, some genes exhibit RNA expression that is not completely in tune with their protein expression. In addition, proteins are more stable than RNA. RNA is prone to degradation within a short period of time or external factors, such as the storage of liver tissues, can affect RNA expression data. Clinical data from patients with HCC, such as tumor size, tumor number, tumor distribution, and preoperative AFP level, are sparse recorded in TCGA database. To determine the biological suitability and reliability of any clinical biomarker, a full investigation of multicentric human clinical samples is required. In addition, there are large differences in gene expression among different races and individuals around the world. In China, the Chinese Han population accounts for more than 92% of the Chinese population. To date, at the translational level, only one study has reported that high EMI2 expression is a poor prognostic factor in patients suffering from breast cancer.^18^ Taken together, at the level of translation, the expression profile of *FBXO43* and its prognostic roles in Chinese Han patients with HCC warrant further investigation.

Our WB results confirmed that FBXO43 protein expression was upregulated in HCC tissues compared with that in paired adjacent normal liver tissues. Subsequently, we analyzed the expression of FBXO43 protein in 93 pairs of HCC tissues and adjacent normal liver tissues using TMA. The proportion of FBXO43‐positive expression in HCC specimens was much higher than that in the adjacent normal liver tissues. High expression of the FBXO43 protein was strongly associated with large tumor size, advanced TNM stage, lymphatic invasion, distant metastasis, and tumor recurrence. The expression level of the FBXO43 protein gradually increased as HCC progressed to a more advanced AJCC stage, which was similar to the trend observed for *FBXO43* RNA expression. Therefore, at the translational level, these findings indicate that *FBXO43* overexpression was related to the clinical progression of HCC. A recent study revealed the role of *FBXO43* in the progression of breast cancer (BC) through a series of in vivo and in vitro experiments.^12^ The proliferation, migration, and invasion capabilities of human BC cells are inhibited by *FBXO43* knockdown.^12^

FBXO7 is located in both the cytoplasm and nucleus.^22^ The FBXO43 protein also appeared in these locations in the human hepatocytes evaluated in our study, which has rarely been reported. Furthermore, regarding the 93 patients with HCC, upregulated expression of the FBXO43 protein was associated with decreased OS and a much earlier carcinoma recurrence after radical surgery. Then, univariate and multivariate regression analyses revealed that the tumor TNM stage, degree of histological differentiation and FBXO43 expression were independent prognostic factors affecting OS and DFS. The results, at both the transcriptional and translation levels, showed a consistent trend regarding the expression profile and prognostic role of FBXO43 in patients with HCC.

The top three genes positively co‐expressed with FBXO43, including *NUF2*,^23^ *NCAPG*,^24^ and *HJURP* ^25^ were oncogenes in HCC. Silencing of *NUF2* in HepG2 human HCC cells can dramatically hampered tumor growth in vivo. Moreover, *NUF2* silencing can induce cycle arrest and trigger cell apoptosis.^26^ *NCAPG* can promote the proliferation of HCC cells through PI3K/AKT signaling,^24^ and over expression of *NCAPG* is associated with poor prognosis in HCC patients with vascular invasion.^27^ High *HJURP* expression indicated poor prognosis in patients with HCC. HJURP overexpression can accelerate the proliferation HCC cells, while *HJURP* knockdown can attenuate the proliferation.^28^ Therefore, we hypothesized that *FBXO43* and its co‐expressed genes may play a synergistic role in promoting the initiation and development of HCC.

Regarding the biological function of FBXO43, FBXO43, also called EMI2, was originally identified in a yeast two‐hybrid screen as a Plx1 (novel polo‐like kinase), and was shown to regulate cell cycle progression in Xenopus eggs.^29^, ^30^ EMI2 as an anaphase‐promoting complex/cyclosome (APC/C) inhibitor regulated by Plx1, is crucial for cytostatic factor (CSF) activity.^15^, ^18^, ^31^ Specifically, APC/C is a well‐known crucial regulator of multiple cellular processes.^14^, ^31^ FBXO43 belongs to the FBXO subclass of the FBP family. FBPs function as tumor promoters and suppressors and participate in other biochemical processes, such as cell cycle regulation, DNA damage repair, and metabolic regulation.^13^, ^14^, ^15^, ^32^ *FBXO7* is a potential oncogene that inhibits apoptosis.^22^ Moreover, FBP is the core component of SKP1‐cullin 1‐F‐box (SCF)‐type E3 ubiquitin ligase.^15^ E3 ubiquitin ligases play an essential role in the molecular mechanisms of tumor progression.^15^ Notably, inhibitors targeting FBPs have shown promising therapeutic potential.^14^

To further elucidate the role of *FBXO43* in HCC, the function and pathway enrichment analysis was performed. The results showed that *FBXO43* and its co‐expressed genes were mainly involved in DNA replication, cell cycle checkpoint, mitotic cell cycle phase transition, Fanconi anemia pathway, homologous recombination, and so on. It was consistent with previously research.^15^ The key roles of F‐box proteins largely depend on their abilities involved in cancer hallmark pathways, including cell cycle, epithelial‐mesenchymal transition and so on, which could contribute to tumor growth, proliferation, progression, metastasis, and invasion.^11^, ^15^ These pathways were also known to be involved in tumor cell proliferation.^33^, ^34^ DNA replication and cell cycle disorder are driving forces of carcinogenesis.^35^ Additionally, based on the DepMep database, *FBXO43* knockdown can attenuate SNU387 human HCC cells growth and proliferation. Above‐mentioned evidences might explain following phenomena. In female *FBXO43* knockout mice, oocytes showed defective entry into meiosis II.^36^ Similarly, in male *FBXO43* knockout mice, spermatocytes fail to complete meiotic divisions.^36^ Even a single homozygous mutation in the *FBXO43* gene can result in infertility, teratozoospermia,^37^ and non‐obstructive azoospermia in male humans.^38^ Several variants of the *FBXO43* gene can lead to early embryonic arrest in female patients.^39^ Knockdown of the *FBXO43* gene decrease cell viability and proliferation in breast cancer cells,^12^, ^17^ suggesting a pro‐tumorigenic role. Overall, *FBXO43* may play an unfavorable role in the early occurrence, the progression, and long‐term prognosis of tumors.

This study had some limitations. First, this was a single‐center study with a small sample size and a relatively high rate of lost to follow‐up, which may affect the accuracy of some results, although we strictly limited the inclusion criteria for HCC patients to avoid unexpected bias. Second, although we have preliminarily investigated the expression characteristics and clinical significance, biological functions, and the underlying mechanism of its involvement of *FBXO43*, the roles of *FBXO43* deserve further verification by other molecular biology experiments and animal experiments. These scientific questions about the *FBXO43* have not been fully answered. Therefore, future studies should be conducted to explore the role and mechanism of *FBXO43* and its products in HCC and even other malignant tumors.

## 5 CONCLUSIONS

Our study revealed that high expression of *FBXO43* RNA or protein predicted a higher risk of HCC, decreased OS, and earlier carcinoma recurrence. Therefore, FBXO43 may be an independent prognostic biomarker in patients with HCC. FBXO43 is worth investigating as a potential HCC treatment target.

## AUTHOR CONTRIBUTIONS

Yiyu Shen mainly contributed to the conception and design of the study. All experiments, acquisition of data, and analysis and interpretation of data were performed by Shaohan Wu, Lei Qin, Juqin Yang, and Jing Wang. Shaohan Wu and Lei Qin drafted the manuscript. All authors commented on the previous versions of the manuscript. All authors have read and approved the final manuscript.

## FUNDING INFORMATION

This study was funded by the Science and Technology Program of Jiaxing (grant number: 2021AD30110).

## CONFLICT OF INTEREST STATEMENT

None.

## Data Availability

The data that support the findings of this study are available from the corresponding author upon reasonable request.
